# Uptake of and Resistance to the Antibiotic Berberine by Individual Dormant, Germinating and Outgrowing *Bacillus* Spores as Monitored by Laser Tweezers Raman Spectroscopy

**DOI:** 10.1371/journal.pone.0144183

**Published:** 2015-12-04

**Authors:** Shiwei Wang, Jing Yu, Milomir Suvira, Peter Setlow, Yong-qing Li

**Affiliations:** 1 Department of Physics, East Carolina University, Greenville, North Carolina 27858–4353, United States of America; 2 Department of Molecular Biology and Biophysics, UConn Health, Farmington, Connecticut 06030–3305, United States of America; Beijing Forestry University, CHINA

## Abstract

Berberine, an alkaloid originally extracted from the plant *Coptis chinensis* and other herb plants, has been used as a pharmacological substance for many years. The therapeutic effect of berberine has been attributed to its interaction with nucleic acids and blocking cell division. However, levels of berberine entering individual microbial cells minimal for growth inhibition and its effects on bacterial spores have not been determined. In this work the kinetics and levels of berberine accumulation by individual dormant and germinated spores were measured by laser tweezers Raman spectroscopy and differential interference and fluorescence microscopy, and effects of berberine on spore germination and outgrowth and spore and growing cell viability were determined. The major conclusions from this work are that: (1) colony formation from *B*. *subtilis* spores was blocked ~ 99% by 25 μg/mL berberine plus 20 μg/mL INF55 (a multidrug resistance pump inhibitor); (2) 200 μg/mL berberine had no effect on *B*. *subtilis* spore germination with L-valine, but spore outgrowth was completely blocked; (3) berberine levels accumulated in single spores germinating with ≥ 25 μg/mL berberine were > 10 mg/mL; (4) fluorescence microscopy showed that germinated spores accumulated high-levels of berberine primarily in the spore core, while dormant spores accumulated very low berberine levels primarily in spore coats; and (5) during germination, uptake of berberine began at the time of commitment (T_1_) and reached a maximum after the completion of CaDPA release (T_release_) and spore cortex lysis (T_lysis_).

## Introduction

Gram-positive spore-forming bacteria of various *Bacillus* and *Clostridium species* have long been of significant research interest, since spores of some of these species cause food spoilage and foodborne diseases, spores of *Bacillus anthracis* are a potential bioterrorism agent, and spores of *Clostridium difficile* are an agent for serious lower gastrointestinal disease [1]. These spores can remain dormant for long periods and are extremely resistant to a variety of environmental stresses. Indeed, antibiotics and many disinfectants commonly used in hospital settings do not readily kill dormant spores. Under appropriate conditions spores can rapidly return to life in the process of germination followed by outgrowth and subsequently cause deleterious effects, but during this process become relatively easy to kill. Thus, a potential strategy to minimize potential hazards of germinated spores is to kill spores once they germinate or during germination.

Detailed knowledge of spore germination biology has come mainly from the study of germination of spores of the model organism *Bacillus subtilis*, and germination of spores of other *Bacillus* and *Clostridium* species has many similarities with that of the model organism [1–8]. Germination of *B*. *subtilis* spores can be triggered by the binding of one or multiple nutrient germinants, L-valine is one, to spore inner membrane proteins called germinant receptors (GRs), leading to a series of events taking place in a defined order. First, a nutrient germinant-GR interaction commits spores to germinate, even if the germinant is removed or displaced from its cognate GRs. This commitment step is likely coincident with the beginning of rapid release of monovalent cations and initiation of slow release of 15–30% of the spore core’s large pool of the 1:1 chelate of pyridine-2,6-dicarboxylic acid (DPA) with divalent cations, predominantly Ca^2+^ (CaDPA). This slow CaDPA release is followed by rapid release of all remaining CaDPA, then degradation of the spores’ peptidoglycan cortex by cortex-lytic enzymes (CLEs) leading to the swelling of the spore core and much water uptake leading to initiation of metabolism [5]. The process of an individual spore’s germination has been divided into four phases according to a spore’s optical intensity in differential interference contrast (DIC) or phase contrast microscopy, with the different phases ending at T_1_, T_lag_, T_release_ and T_lysis_. T_1_ is the time between the addition of germinant and the start of slow CaDPA leakage and is probably coincident with the time of commitment, T_lag_ is the time when rapid CaDPA release begins, T_release_ is the time when rapid CaDPA release is completed. Following T_release_, there is a further decline in spore refractility due to the hydrolysis of the spore cortex and spore core swelling, and T_lysis_ is the time when spore refractility becomes constant.

Berberine is a well-known isoquinoline alkaloid originally extracted from a number of plant species, including those of the genera *Berberis*, *Mahonia* and *Coptis* [9]. Berberine has been widely used as a broad-spectrum anti-microbial medicine for over 3000 years in China and used for wide clinical applications in Native American and Western medicine [10]. Berberine has been shown to exhibit activity against viruses, fungi, protozoans, helminths and a variety of bacteria, including many pathogenic species and multidrug resistant strains of *Mycobacterium tuberculosis* and methicillin resistant *Staphylococcus aureus*. Berberine’s antibacterial activity has been ascribed to effects on cell membranes, interactions with DNA, and inhibition of cell division [11–13]. However, the effects of berberine on spores of pathogenic spore-forming bacteria have not been well studied. Prior work has found that berberine at high concentrations inhibits the growth of *B*. *subtilis* vegetative cells [14]. However, the effects of berberine on *B*. *subtilis* spores, especially when berberine is combined with inhibitors of multidrug resistance pumps (MDRs) that can pump berberine out of cells and thus enhance bacterial resistance to this compound, are not clear. In addition, berberine levels accumulated in single cells and the kinetics of berberine uptake in cells have not been determined for any bacterium. In this study, laser tweezers Raman spectroscopy (LTRS) in combination with fluorescence and DIC microscopy were used to analyze the location, levels and uptake kinetics of berberine in individual dormant and germinated *B*. *subtilis* spores. The effects of combinations of berberine and an MDR inhibitor, INF55, on spore germination and outgrowth were also examined.

## Materials and Methods

### Strain used and spore preparation

Berberine chloride and the MDR inhibitor INF55 were purchased from ChemBridge (San Diego, CA). The *B*. *subtilis* strain PS533, a derivative of a laboratory 168 strain that carries plasmid pUB110 providing resistance to kanamycin (10 μg/mL), was used [15]. Spores of *B*. *subtilis* were prepared at 37°C on 2× Schaeffer’s-glucose medium agar plates and were purified as described previously [16]. All spores used in this study were > 98% free of sporulating cells, germinated spores and debris as observed by phase contrast microscopy.

### Effects of berberine on spore germination, outgrowth and colony formation

Spores were routinely heat activated in water before germination by incubation at 70°C for 30 min and then cooling on ice for at least 15 min. Spore germination was performed in 10 mM L-valine in K-HEPES buffer (pH 7.4) with spores at a concentration of ~10^8^/mL unless stated otherwise, but in some experiments germination was conducted in LB medium containing 5 or 10 mM L-valine and plus or minus 25 mM K-HEPES buffer (pH 7.4) [7,8,17].

Two methods were used to measure effects of berberine on colony formation from spores: (a) heat-activated spores at ~5×10^8^/mL were serially diluted in water, 100 μL aliquots (~ 300 spores) spread on LB medium agar plates with different concentrations of berberine with or without 20 μg/mL INF55, plates incubated at 37°C for 12–18 h, and colonies were counted; and (b) heat-activated spores (~10^7^/mL) were incubated for 1 h at 37°C with 10 mM L-valine in 25 mM K-HEPES buffer (pH 7.4) plus different concentrations of berberine with or without 20 μg/mL INF55, spores serially diluted in water, 100 μL aliquots (~300 spores) spread on LB plates without berberine and INF55, plates incubated at 37°C for 12–18 h, and colonies were counted. Incubation for longer times gave no increase in the numbers of colonies using either method.

Effects of berberine on spore germination and outgrowth were also determined by two methods. In one heat-activated spores were incubated as described above at 37°C in LB medium plus 5 mM L-valine and without or with berberine (200 μg/mL). Beginning at T_0_, the optical density at 600 nm (OD_600_) of the incubations was measured every 15 min to monitor germination and outgrowth, since as DPA is released and the cortex is hydrolyzed the OD_600_ falls; in contrast, in outgrowth and subsequent vegetative growth the OD_600_ rises. In the second method, the germination of multiple individual heat-activated spores adhered on a microscope slide and incubated at 37°C in 10 mM L-valine and 25 mM K-HEPES buffer (pH 7.4) at 37°C was monitored by DIC microscopy [7,17].

### Levels of berberine uptake and its location in individual spores

For fluorescence microscopy to localize berberine in spores, heat-activated dormant spores were incubated at 23°C for 60 min in 25 mM K-HEPES buffer (pH 7.4) with or without berberine (50 μg/mL), and spores were germinated with L-valine at 23°C for 60 min as described above. When excited by wavelengths of 460–470 nm, berberine molecules emit strong fluorescence at ~520 nm. Fluorescence images at 520 nm of single spores were obtained with an inverted fluorescence microscope (Olympus IX81) with excitation at 473 nm. Berberine levels in individual spores were determined by LTRS as described previously [7,8]. Briefly, an individual dormant or germinated spore was randomly optically trapped in an aqueous medium with a 780-nm laser beam with a power of 20 mW, and Raman scattering light excited by the same laser beam was measured with a charge-coupled device (CCD) coupled with a spectrograph [7,8]. The spores’ berberine content was determined from the intensity of the berberine-specific Raman band at 1,518 cm^-1^ compared to the intensities of this band from solutions of known concentrations of pure berberine.

### Monitoring spore germination and berberine uptake

In order to determine when berberine was taken up by germinating spores, two methods were used. (a) The kinetics of CaDPA release and berberine uptake during L-valine germination of individual spores at 37°C that were optically trapped by laser tweezers were measured simultaneously by DIC microscopy and Raman spectroscopy as described previously [7,17]. CaDPA and berberine levels in individual trapped spores were determined from the intensities of CaDPA- and berberine-specific Raman bands at 1,017 cm^-1^ and 1,518 cm^-1^, respectively. As found previously [7,17], the end of the rapid fall in spores’ DIC image intensity during spore germination corresponded to the point at which release of CaDPA was complete, and this time was defined as T_release_. At this time, the DIC image intensity (I) was 30 to 35% of that at time zero (T_0_), when image intensity at T_0_ was set at 1 and the DIC image intensity at the end of measurements was set at zero. The CaDPA content of spores at T_0_ was also set at 1 and the content at the end of measurements was set at zero, since the DIC intensity was nearly coincident with the CaDPA level prior to T_release_ [17]. In addition to T_release_, the parameters T_1_, T_lag_ and T_lysis_, described in the Introduction were also used to describe the kinetics of the germination of individual spores. Two additional kinetic parameters that were used were ΔT_leakage_ and ΔT_release_, where ΔT_leakage_ = T_lag_—T_1_, and ΔT_release_ = T_release_—T_lag_. In order to compare CaDPA release and berberine uptake kinetics, the berberine content in spores at the end of measurements was subsequently set at 1. (b) DIC and fluorescence microscopy were also used simultaneously to analyze the kinetics of berberine uptake in multiple individual germinating spores. In brief, spores were spread on the surface of a coverslip that was dried in a vacuum desiccator for ~ 10 min, and the coverslips were mounted on and sealed to a microscope sample holder kept at 37°C with 10 mM L-valine, 35 μg/ml berberine and 25 mM K-HEPES buffer (pH 7.4) [7,17]. The illumination light source for DIC microscopy was replaced by a high power blue light emitting diode (470 nm) in an inverted microscope. A narrow-band pass filter at 520 nm was placed in front of a CCD camera to record the fluorescence images and a band pass filter at 470 nm was placed in the front of a DIC CCD camera to record the DIC images. The DIC and fluorescence images of multiple spores adhered on coverslips were recorded at a rate of 1 frame per 15 s for 60 min by a digital CCD camera (16 bits; 1,600 × 1,200 pixels) and a CCD camera (16 bits, 1,360 × 1,024 pixels) (DS-145M ICE, Opticstar, Manchester, UK). DIC images were analyzed by the Matlab program to locate and label individual spores and to calculate spores’ DIC and fluorescence intensities.

## Results

### Effects of berberine on colony formation of *B*. *subtilis* spores

**Fig 1:**
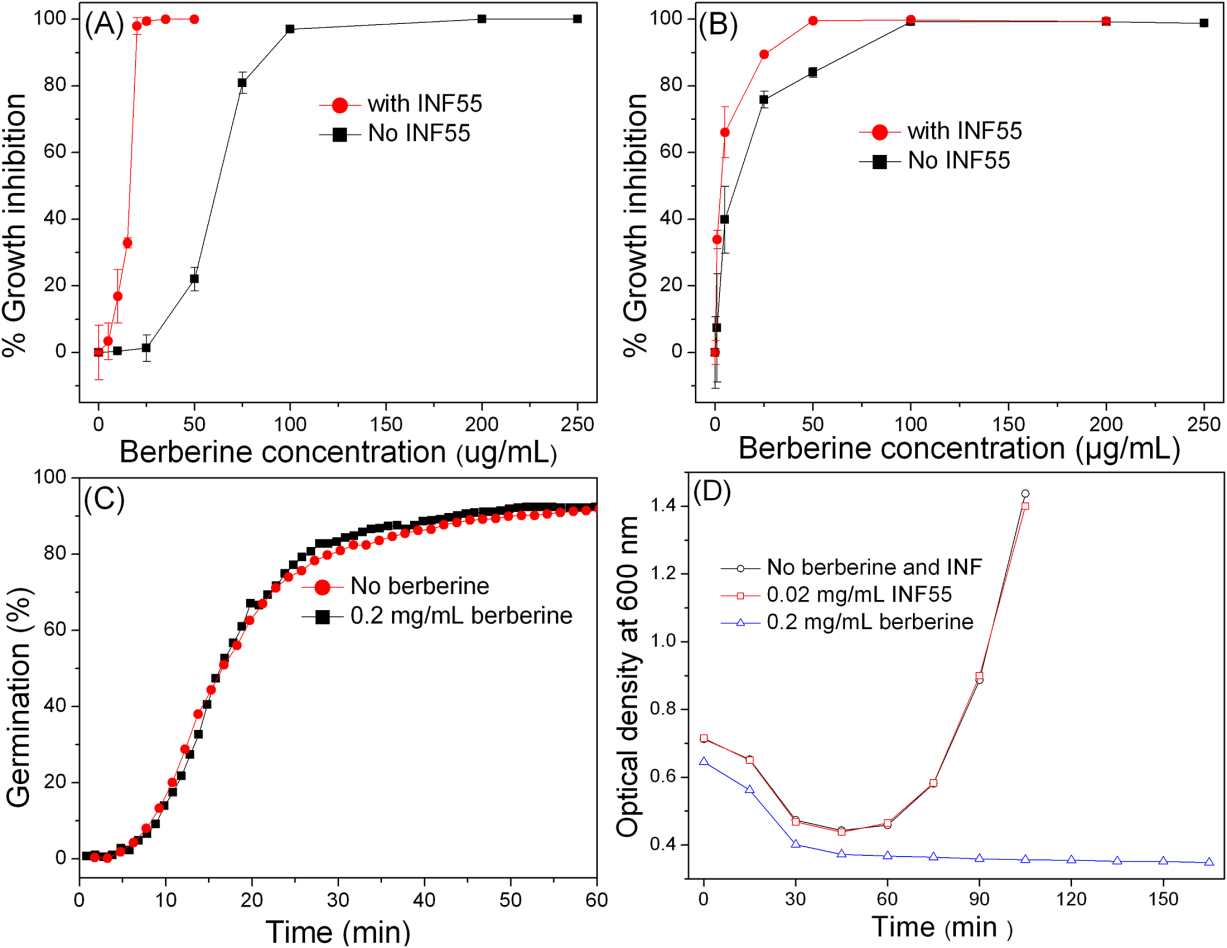
Effects of berberine on spore germination and outgrowth. **(A)** Spores (~300) were directly spread on LB plates containing various berberine concentrations, and with or without 20 μg/mL INF55, and incubated at 37°C for 12–18 h. **(B)** Spores (~10^7^/mL) were first germinated with L-valine for 1 hr at 37°C as described in Methods plus various berberine concentrations, and with or without 20 μg/mL INF55. The germinated spores (~300) were then spread on LB plates without berberine and INF55, plates incubated at 37°C for 12 -18h, and growth was determined as described in Methods. **(C)** Germination of individual spores germinated at 37°C with L-valine and without berberine or with 200 μg/mL berberine. The kinetic germination was determined by the data for > 390 individual spores. **(D)** Germination and outgrowth of PS533 spores in LB medium plus valine, and without berberine or with 200 μg/mL berberine. Heat-activated spores were added at zero time, and the OD_600_ falls as DPA is released and the cortex is hydrolyzed. In the absence of berberine, the germinated spores outgrow and ultimately grow vegetatively leading to an increase in OD_600_; however, with berberine there is no increase in OD_600_ and thus no outgrowth.

Like vegetative cell growth with a berberine [18], colony formation from *B*. *subtilis* spores decreased with increased berberine concentrations in LB plates, and berberine at 100–200 μg/mL almost completely inhibited colony formation (Fig 1A). However, when berberine was combined with 20 μg/mL of the MDR inhibitor INF55, colony formation from spores was inhibited completely at 25 μg/mL berberine (Fig 1A), suggesting that MDRs play a role in protecting *B*. *subtilis* against berberine.

Since germinated spores are much easier to kill than dormant spores, triggering spore germination in the presence of berberine and INF55 might be a way to readily kill spores. To test this strategy spores were germinated with L-valine alone for 1 hr at 37°C as described in Methods and with or without INF55 (20 μg/mL) and various berberine concentrations, spread on LB plates without berberine and INF55, incubated for 12–18 hr at 37°C, and colonies were counted (Fig 1B). The results showed that L-valine germination for 1 hr with berberine and INF55 inhibited almost all colony formation on LB plates, indicating that berberine is bactericidal, although berberine alone was more effective in abolishing colony formation from spores germinating with L-valine than from dormant spores spread on LB medium plates (Fig 1A). In addition, while INF55 enhanced the killing of spores germinated with berberine, INF55 was more effective on dormant spores spread on LB medium plates (Compare Fig 1A and 1B). In addition to killing germinated spores, 200 μg/mL berberine also killed log phase cells growing in LB medium > 99.9% in 30 min (data not shown), indicating that this agent is bactericidal for growing cells as well as germinated spores.

### Effects of berberine on spore germination and outgrowth

The results given above indicated that berberine inhibited colony formation from spores, but this could be by effects on spore germination, outgrowth of germinated spores, or cell growth. To discriminate between these possibilities, spores were germinated with L-valine and with or without 200 μg/mL berberine and the germination of ~ 395 individual spores was monitored by DIC microscopy (Fig 1C). There was clearly no significant difference in the germination of spores with and without this berberine concentration, and the kinetic parameters of the germination of multiple individual spores with and without 200 μg/mL berberine, including average values of various key times in germination were also identical (S1 Fig and S1 Table). Thus berberine clearly has no effects on spore germination.

To test whether berberine has effects on spore outgrowth, we also measured germinated spores’ ability to swell and elongate by monitoring the OD_600_ of cultures germinating with L-valine in LB medium, and with or without 200 μg/mL berberine (Fig 1D). Again, the fall in OD_600_ reflecting spore germination was identical in spores incubated in LB medium +/- berberine. However, while the OD_600_ then increased dramatically in the culture without berberine, reflecting spore outgrowth and ultimately vegetative growth, the spores incubated with berberine never exhibited an increase in their OD_600_. This result thus indicates that while berberine does not inhibit spore germination, it does inhibit germinated spores’ outgrowth.

### Levels and location of berberine in spores

**Fig 2:**
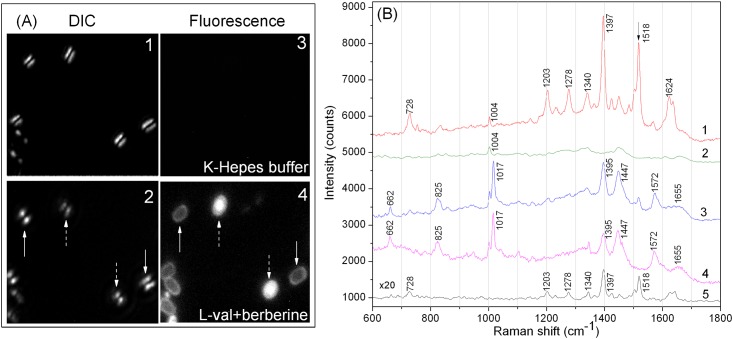
Location and identification of berberine in spores. **(A)** DIC images and fluorescence images of individual dormant spores (panes 1, 3), or germinated spores that were incubated in 10 mM L-valine at 23°C for 60 min as described in Methods plus 50 μg/mL of berberine (panes 2, 4). Note that a single spore appears as two bright spots in the DIC image due to the differential interference of illumination light. Berberine fluorescence at 520 nm was acquired with excitation at 473 nm. Germinated spores (dashed arrows) appeared dim in DIC microscopy but bright in fluorescence microscopy, and ungerminated spores (solid arrows) were bright in DIC microscopy but dim in fluorescence microscopy. **(B)** Average Raman spectra of 30 individual spores that were: curve 1—germinated as described in panel (A) with 200 μg/mL berberine; curve 2—germinated as described in panel (A) without berberine; curve 3—ungerminated after incubation at 37°C for 60 min in 25 mM K-HEPES buffer (pH 7.4) and 200 μg/mL berberine; and curve 4—dormant and incubated as described for curve 3 without berberine. Curve 5 is a Raman spectrum of 200 μg/mL berberine in 25 mM K-HEPES buffer (pH 7.4). The peak intensities in curve 5 were multiplied by 20.

Since berberine is able to inhibit spore outgrowth, obvious questions are how much berberine has actually entered outgrowing spores and where is this berberine located. Because berberine can fluoresce upon proper excitation, we initially used fluorescence microscopy to localize berberine in spores. As was not surprising, berberine was readily detected in germinated spores, and in spores’ central region or core where DNA and RNA is located (Fig 2A). However, there were minimal levels of berberine in dormant spores, and this berberine was primarily in spores’ outer layer, most likely adsorbed to the spore coat (Fig 2A). As expected, there were minimal levels of fluorescence in dormant spores incubated in buffer without berberine (data not shown).

In order to quantitate berberine levels in spores, Raman spectroscopy was first used to compare Raman spectra of dormant and germinated spores with or without berberine, as well as berberine alone (Fig 2B). The two largest Raman spectral peaks from berberine alone were at 1,397 and 1,518 cm^-1^ (Fig 2B, curve 5). As seen previously, dormant spores alone gave multiple Raman peaks, with major ones at 1,017, 1,395, 1,447 and 1,572, all due to the high levels of CaDPA in the spore core (Fig 2B, curve 4) [10,14,16,17]. Germinated spores without berberine lacked peaks due to CaDPA (Fig 2B, curve 2), since spores’ large CaDPA depot (~ 20% of spore core dry wt) is excreted in the first min of spore germination [6]. Consistent with the fluorescence microscopy results, the average Raman spectra of 30 individual spores germinated with berberine had large berberine-specific peaks at 1,397 and 1,518 cm^-1^, while the average Raman spectra of 30 individual dormant spores incubated with berberine had very small peaks at 1,397 and 1,518 cm^-1^ (Fig 2B, curves 1 and 3, respectively), suggesting that while there is much berberine in germinated spores, there are only minimal levels in dormant spores.

### Berberine levels in individual spores germinated in a rich medium

**Fig 3:**
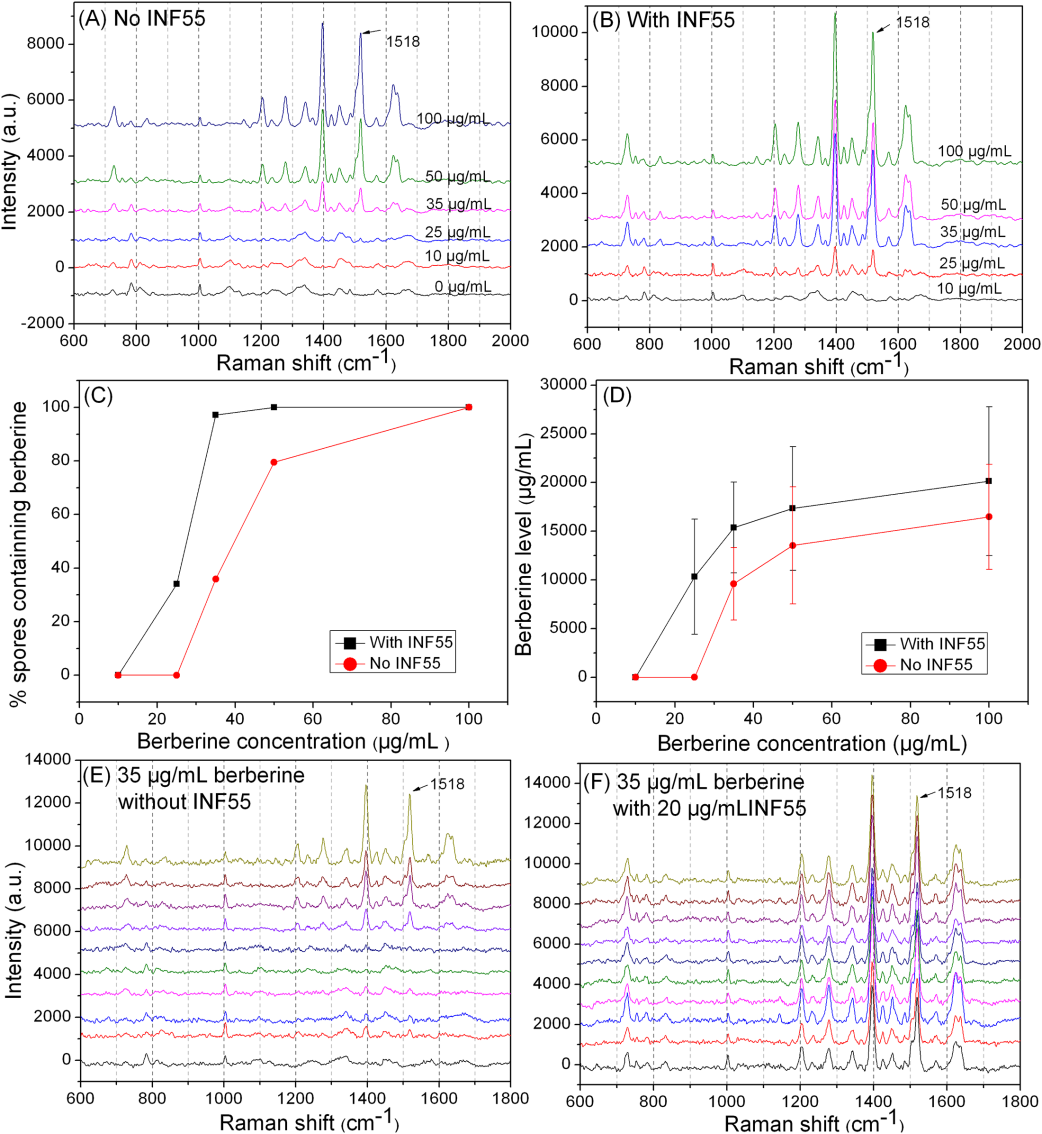
Berberine levels accumulated by germinated spores as determined by LTRS. **(A, B)** Average Raman spectra of 50 individual spores germinated with various berberine concentrations for 60 min at 37°C in LB medium plus 10 mM L-valine without (A) or with (B) 20 μg/mL INF55. **(C)** The percentage of germinated spores that contain berberine. **(D)** The average berberine level in germinated spores that contained berberine. Berberine levels were determined by the Raman band intensities at 1,518 cm^-1^ in spores, calibrated by Raman band intensities of berberine solutions of known concentration. **(E, F)** Raman spectra of 10 individual spores germinated as described above with 35 μg/mL berberine without (E) or with (F) 20 μg/mL INF55.

In order to germinate spores quickly, spores were incubated in LB medium plus L-valine and various berberine concentrations, and berberine levels of individual spores were determined by LTRS (Fig 3). With berberine concentrations of 10 to 25 μg/mL and without INF55, there was no berberine in germinated spores, but when berberine concentrations were increased to ≥ 35 μg/mL the percentage of spores containing berberine and their berberine level gradually rose (Fig 3A and 3C, Table 1). In the presence of 20 μg/mL INF55 and 10 μg/mL berberine concentration, there was still no berberine in germinated spores (Fig 3B and 3C). However, when berberine concentrations were ≥ 25 μg/mL the percentage of spores containing berberine and their berberine level gradually rose (Fig 3B) and these values were more than those at the same berberine concentrations but without INF55 (Fig 3C and 3D). For example, when berberine concentration was 35 μg/mL without INF55, only ~ 40% of the germinated spores contained significant amounts of berberine. However, with the same berberine concentration of 35 μg/mL plus INF55 all germinated spores contained berberine (Fig 3E and 3F). This is presumably the reason that treatment with low berberine concentrations only gave partial inhibition of colony formation from spores on LB plates even with 20 μg/mL INF55 present, suggesting that MDRs of most germinated spores were able to rapidly excrete berberine taken up at moderate berberine concentration.

**Table 1:** Berberine levels in single spores germinated with various berberine concentrations and with or without INF55*.

Valine germination in	Berberine concentration during germination (μg/mL)	Percentage of spores containing berberine (with INF55) (%)	Berberine level in germinated spores (with INF55) (mg/mL)	Ratio of berberine level inside cell/outside cell (with INF55)
LB medium	10	0 (0)	0 (0)	0 (0)
LB medium	25	0 (34)	0 (10.3±5.9)	0 (413±237)
LB medium	35	36 (97)	9.6±3.7 (15.3±4.7)	274±106 (439±133)
LB medium	50	80 (100)	13.5±6.0 (17.3±6.4)	271±120 (347±127)
LB medium	100	100 (100)	16.5±5.4 (20.2±7.6)	165±54 (202±76)
K-HEPES buffer	10	100 (100)	0.7±0.3 (2.7±0.7)	65±32 (268±72)
K-HEPES buffer	25	100 (100)	2.4±0.5 (3.3±0.8)	95±20 (134±30)
K-HEPES buffer	35	100 (100)	6.1± 1.2 (7.2±1.9)	174±35 (206±55)
K-HEPES buffer	50	100 (100)	9.2±2.2 (9.8±2.4)	184±43 (195±49)
K-HEPES buffer	100	100 (100)	14.0±3.0 (14.4±3.7)	140±30 (144±37)

*Spores were germinated at 37°C for 60 min with 10 mM L-valine in either buffer alone or in buffer plus LB medium, and with various concentrations of berberine and with or without INF55 (20 μg/mL). Berberine levels in germinated spores were determined by Raman spectroscopy as described in Methods, and values for berberine levels in spores are averages of data from ~50 individual spores.

When spores were germinated with 20 μg/mL INF55 and with increasing berberine concentrations, the berberine concentration inside germinated spores rose from 0 to 20.2 mg/mL, and the ratio of berberine concentrations inside to outside spores ranged from 0 to 439 (Table 1). When spores were germinated without INF55 and with increasing berberine concentrations, the berberine concentration in spores increased from 0 to 16.5 mg/mL, and the ratio of berberine concentrations inside and outside spores increased from 0 to 274 (Table 1).

**Fig 4:**
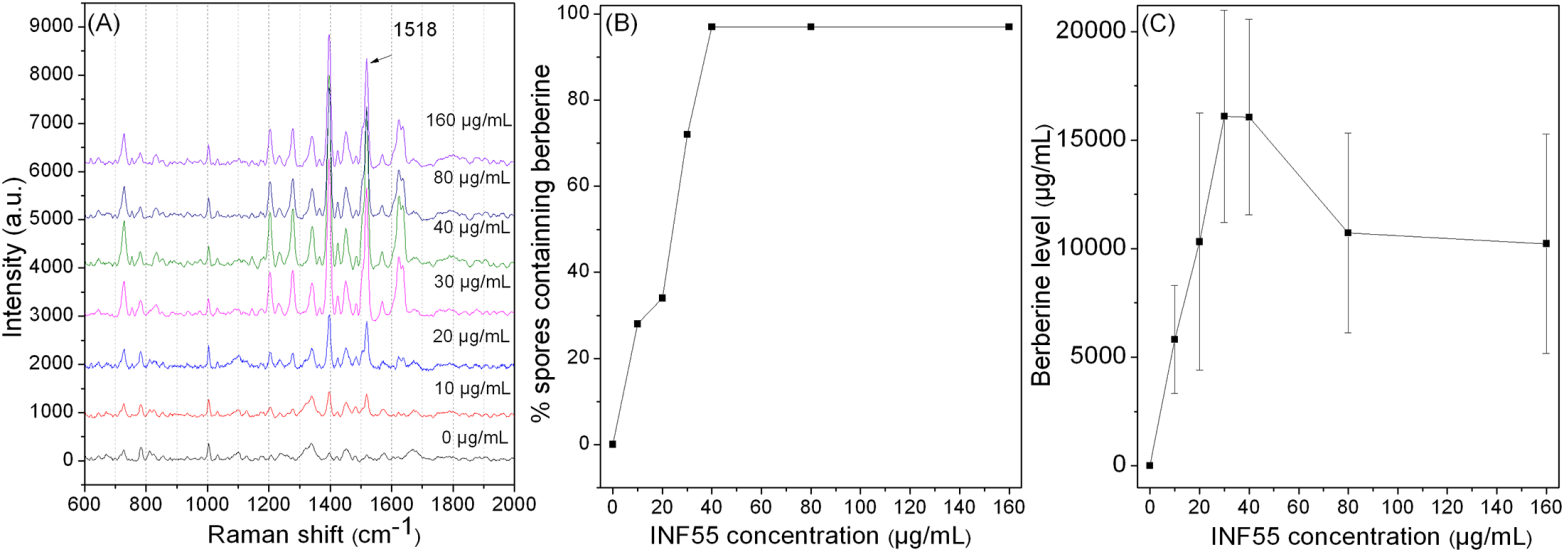
Berberine levels accumulated by spores that were incubated with 25 μg/mL berberine plus various concentrations of INF55 at 37°C for 60 min in LB medium plus 10 mM L-valine. **(A)** Average Raman spectra of 50 individual spores, **(B)** The percentage of germinated spores that contained berberine. **(C)** The average berberine level in germinated spores that contained berberine with various INF55 concentrations and 25 μg/mL berberine. When INF55 concentration was ~ 30 μg/mL, the berberine level accumulated in the germinated spores reached the maximum.

To test if 20 μg/mL INF55 is sufficient to give maximal inhibition of MDR activity, berberine levels were measured when spores were germinated with various INF55 concentrations and 25 μg/mL berberine in LB medium plus L-valine. With INF55 concentrations of 10 to 40 μg/mL, the percentage of spores containing berberine and their berberine level gradually rose to 100% and ~16.5 mg/mL, respectively. However, further increases in the INF55 level did not significantly increase berberine levels (Fig 4).

### Berberine levels in spores germinated with L-valine alone

**Fig 5:**
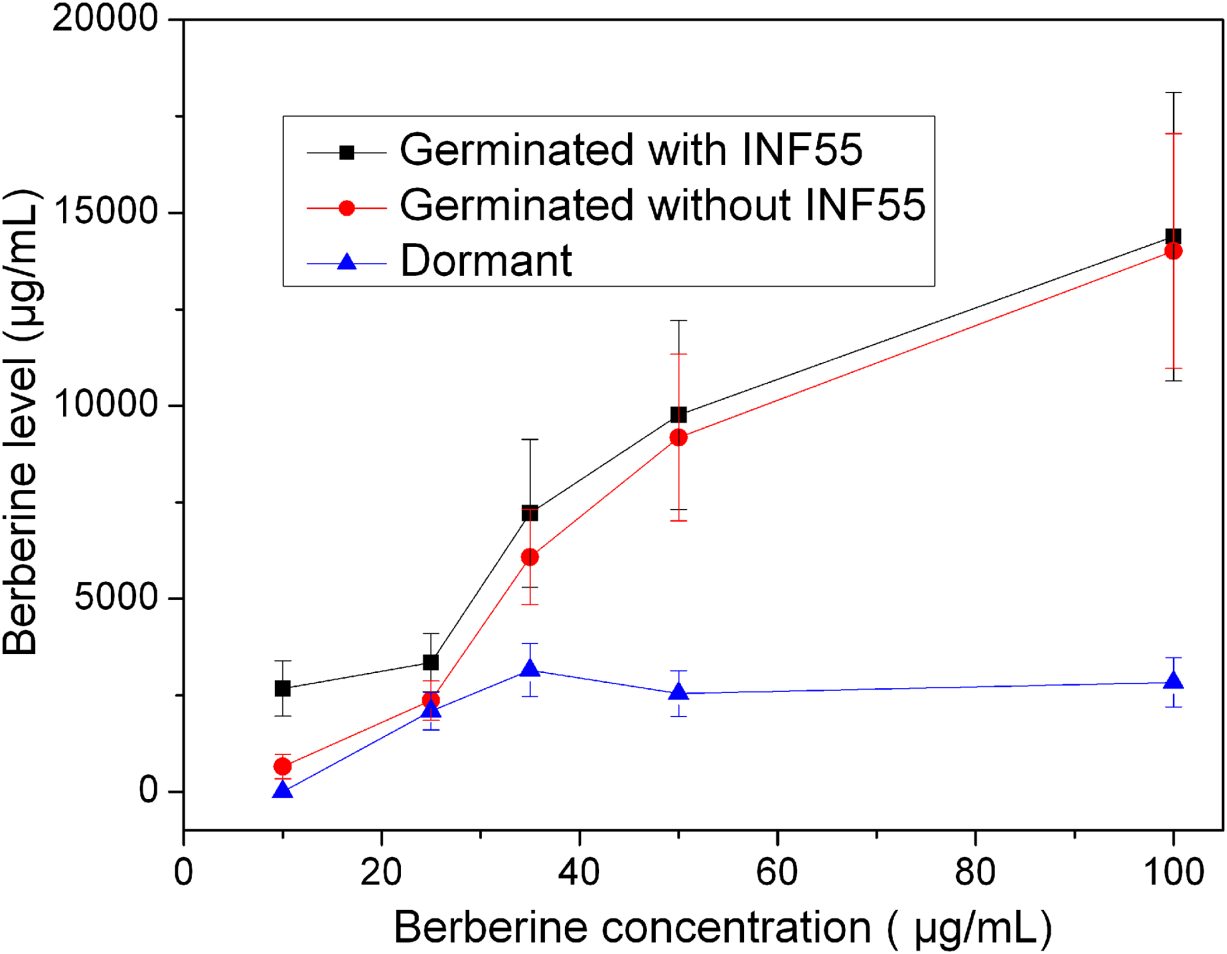
Levels of berberine in single dormant and germinated spores. Spores were incubated for 60 min at 37°C in buffer and different concentrations of berberine without (dormant spores) or with (germinated spores) 10 mM L-valine, and Raman spectra were measured by LTRS. Spores were also incubated similarly in buffer and valine plus 20 μg/mL INF55 before Raman measurements. Berberine levels in spores were calculated as described in Methods. The curves shown are averages of data from ~ 30 individual spores.

The levels of berberine in individual spores were also measured when spores were incubated with various berberine concentrations in buffer and with or without 20 μg/mL INF55 or 10 mM L-valine (Fig 5). As indicated above with berberine concentrations of 50 μg/mL (Fig 2), dormant spores incubated with berberine concentrations up to 100 μg/mL also accumulated berberine although the amounts of berberine seemed to be constant with berberine concentrations from 35 to 100 μg/mL. As seen with spores germinated in a rich medium, the berberine level inside spores germinated with L-valine alone gradually rose with an increase in berberine concentration from 0 to 100 μg/mL reaching almost 14.4 mg/mL. However, in spores germinated with L-valine in buffer, INF55 only had effects on berberine concentrations inside germinated spores at an exogenous berberine concentration of 10 μg/mL (Fig 5). Notably, the high berberine levels accumulated by these germinating spores were maintained for up to 9 hr (data not shown).

### Berberine was taken up into spores late in germination

**Fig 6:**
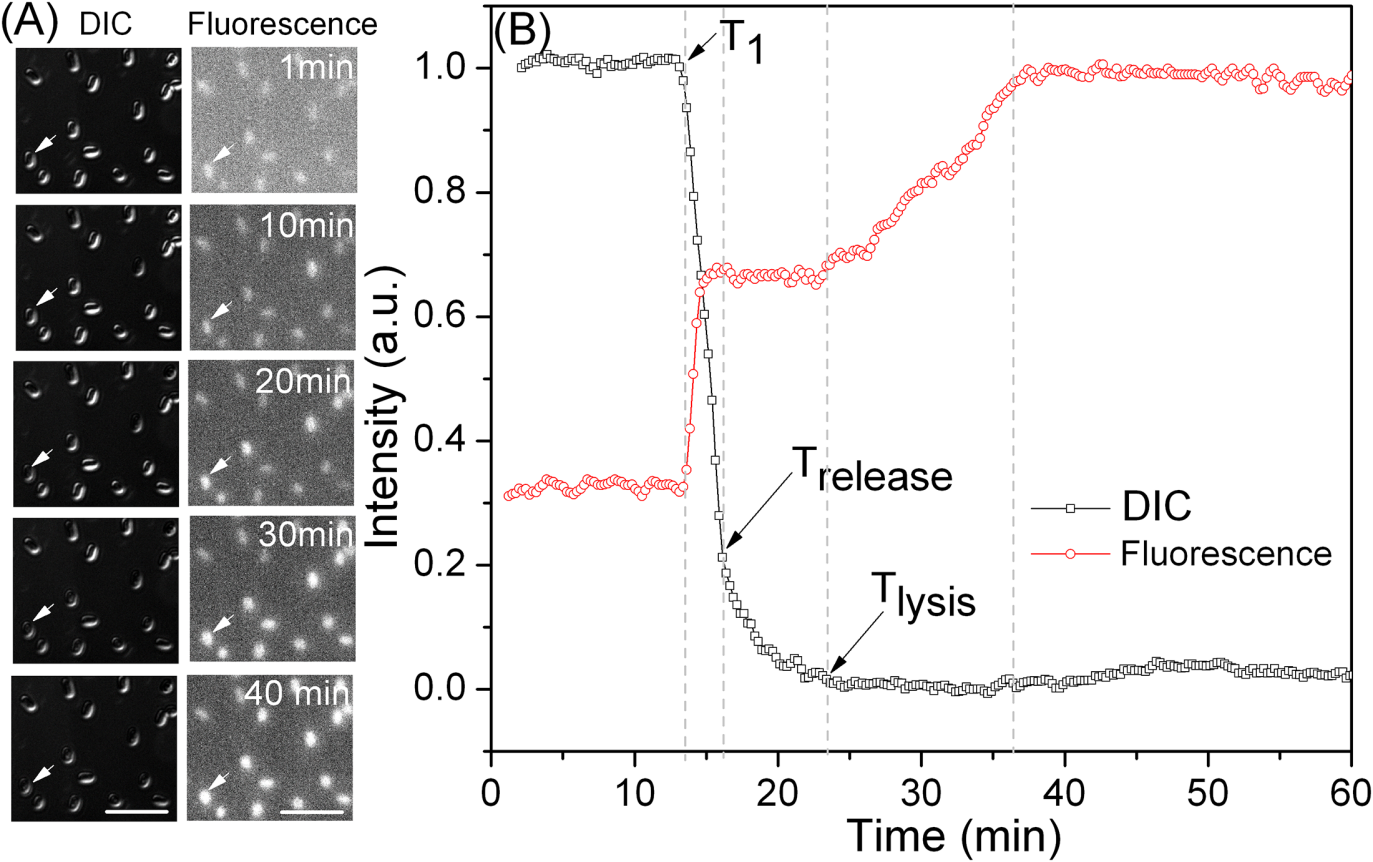
Simultaneous recording of DIC and fluorescence image intensity of individual *B*. *subtilis* spores adhered on a coverslip germinating at 37°C with 10 mM L-valine and 35 μg/mL berberine in 25 mM K-HEPES buffer. **(A)** Sequential DIC and fluorescence images during spore germination. The scale bar in (A) is 5 μm. **(B)** DIC and fluorescence image intensities as a function of germination time. The arrows indicate the times of T_1_, T_release_ and T_lysis_. DIC image intensities were calculated by normalizing a spore’s DIC image intensity to its initial value at the first time of measurement (corresponding to that of the dormant spore) after subtraction of the last unchanged image intensity value (corresponding to that of the fully germinated spore). The fluorescence image intensities were normalized to the maximum intensity value. All intensities are given in arbitrary units (a.u.).

While spore germination was not inhibited by berberine, spore outgrowth certainly was, and germinated spores accumulated high berberine levels. An obvious question then is when is berberine taken up by germinating spores? To answer this question, DIC and fluorescence microscopy were combined to simultaneously observe spore germination and berberine levels. When spores adhered on coverslip were exposed to 10 mM L-valine and 35 μg/mL berberine in buffer, some berberine was taken up almost immediately, presumably reflecting berberine adsorbtion to spores’ outer layer, and this level did not change until T_1_ when more berberine was quickly taken up (Fig 6). Notably, this uptake ended prior to T_release_, and again, there was no change until T_lysis_ when there was further berberine uptake. Similar results were observed for 6 other individual germinating spores, although the different spores exhibited significant variation in the amounts of berberine taken up into spores at the three phases noted above (S2 Fig).

**Fig 7:**
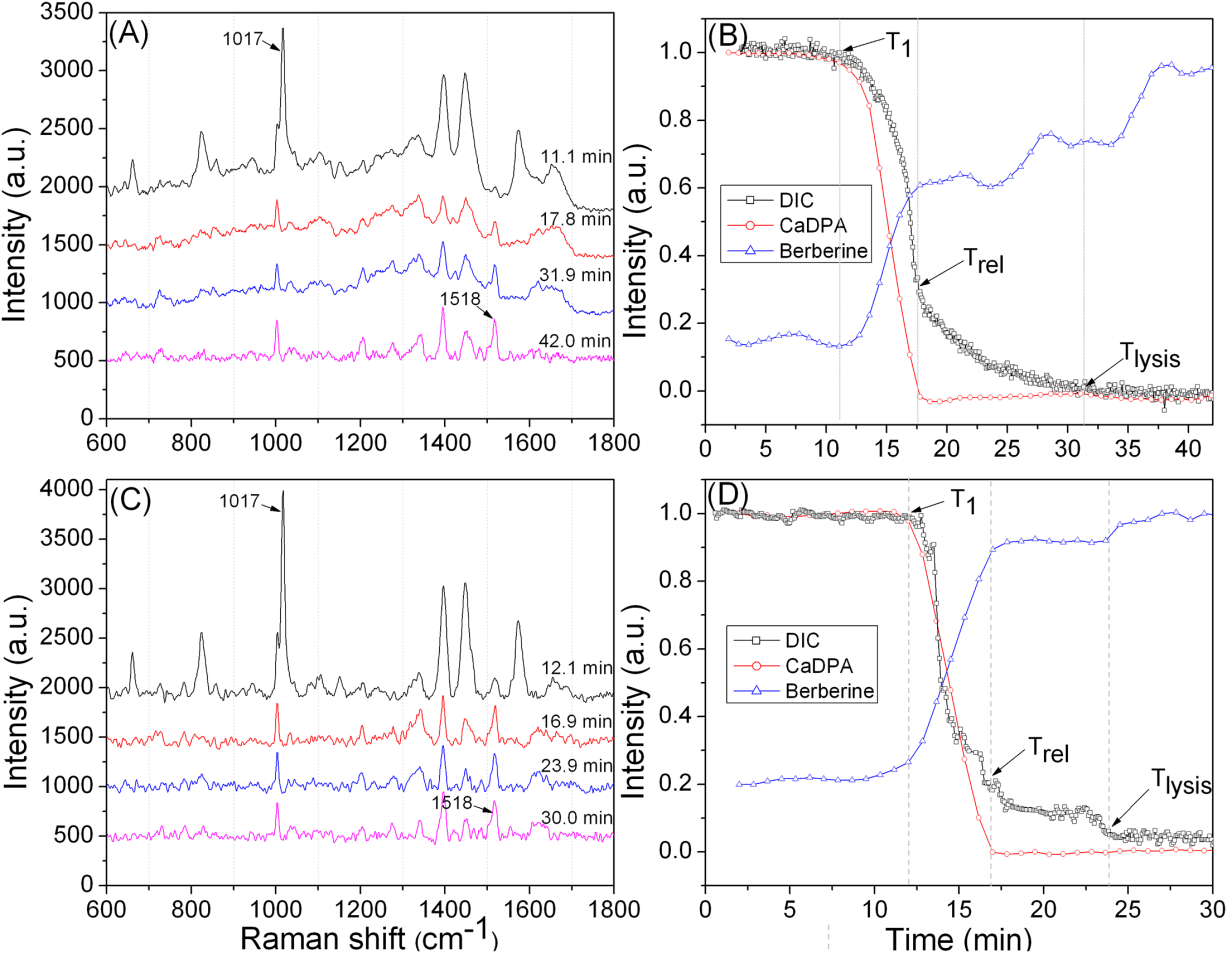
Simultaneous DIC image intensities and Raman spectra of single optically trapped *B*. *subtilis* spores germinating with berberine. **(A, C)** Time-lapse Raman spectra of two individual spores germinating at 37°C with 10 mM L-valine and 35 μg/mL berberine in 25 mM K-HEPES buffer. **(B, D)** Intensities of CaDPA and berberine-specific Raman bands at 1,017 cm^-1^ and 1,518 cm^-1^ and DIC image intensity as a function of germination time. The black arrows indicate the times of T_1_, T_release_ and T_lysis_. The DIC image intensities were normalized to its initial value at the first time of measurement (corresponding to that of the dormant spore) after subtraction of the last unchanged image intensity value (corresponding to that of the fully germinated spore). CaDPA-band intensities at 1,017 cm^-1^ were normalized to the initial values at the first time of measurement, and berberine-band intensities at 1,518 cm^-1^ were normalized to the maximum intensity value, as described in Methods.

In order to confirm that the fluorescence observed in spores is indeed due to berberine, laser tweezers were used to capture an individual spore, L-valine and berberine added, and DIC microscopy and Raman spectroscopy were used to simultaneously monitor CaDPA release, berberine uptake and spore cortex lysis (Fig 7). Similar to what was observed by DIC and fluorescence microscopy, the results showed that berberine uptake was minimal prior to T_1_ of germination, became significant between T_1_ and T_release_, stopped soon after T_release_, and then increased after T_lysis_.

## Discussion

Previous work has shown that berberine blocks growth of both Gram negative and Gram positive bacteria including *B*. *subtilis*, and the mechanisms of berberine action include both binding to nucleic acids as well as inhibiting cell division by binding to the crucial cell division protein FtsZ [11–14,19]. In the current work we have confirmed that berberine inhibits *B*. *subtilis* cell growth and have shown that berberine is also bactericidal. However, berberine did not inhibit spore germination at all, presumably because this agent did not penetrate the dormant spore core, and this site of spore nucleic acids and cell division is not required for spore germination. In contrast, spore outgrowth was completely inhibited by berberine, with no evidence of even any elongation of germinated spores following germination in the presence of berberine. This latter observation as well as the rapid berberine uptake into the spore core beginning when CaDPA was rapidly released in spore germination indicates that there may be minimal if any protein and probably RNA synthesis in spores germinated with berberine. Since cell division following spore germination does not take place for many min and well after the germinated spore has elongated due to macromolecular synthesis [1,20], these observations indicate that berberine exerts toxic effects on the germinated spore by interaction with spore nucleic acids, presumably DNA and not by blocking cell division. However, it is possible that lower berberine levels than used in the outgrowth experiment would have allowed some macromolecular synthesis but blocked cell division.

As noted above, berberine was taken up rapidly early in the germination process and approximately in parallel with the rapid CaDPA release by a germinating spore. Previous work has studied the kinetics of the uptake of the nucleic acid binding dye Syto 16 by germinating *B*. *cereus* spores [21]. This work found that Syto 16 uptake is minimal until after rapid release of all remaining CaDPA is almost complete, and takes place primarily during the time of spore cortex hydrolysis between T_release_ and T_lysis_. Thus berberine uptake in spore germination significantly precedes Syto 16 uptake. This could be because the berberine molecule (mol wt 336) is smaller than that of Syto 16 (mol wt ~ 450), or because of differences in the charge or hydrophobicity of these two molecules. However, the precise mechanism for the uptake of berberine by germinating spores is not known, although this seem likely to be an ATP-independent mechanism, since significant ATP production by germinating spores does not take place until after spore cortex hydrolysis [1], well after significant berberine uptake by germinating spores.

While berberine was effective in killing germinated spores, this required rather high berberine concentrations. Previous work has indicated that berberine is normally expelled rapidly from cells by various MDRs [18,22]. This also seems to be the case with germinating spores, as MDR inhibitors decreased the berberine concentrations needed for inhibition of cell growth from dormant or germinated spores. It was, however, notable that the MDR inhibitor INF55 was much more effective in potentiating berberine effects on germinated spores if spore germination was carried out in a complete nutrient medium, rather than in buffer plus L-valine alone. It certainly seems likely that much less ATP is generated in spores that are germinated in L-valine alone rather than in a complete nutrient medium such as LB broth. Consequently, since berberine efflux by MDRs requires ATP, either directly or indirectly, much less ATP is available for MDR use in pumping out berberine from spores germinated in L-valine alone and thus MDRs are less effective in these spores allowing berberine alone to be more effective in killing the germinated spores.

Obvious questions about berberine efflux in germinated *B*. *subtilis* spores as well as growing cells are the identity of MDR efflux proteins, whether these MDR proteins are present in dormant *B*. *subtilis* spores, and whether these proteins are synthesized early in spore outgrowth. A number of MDR proteins have been identified in *B*. *subtilis*, and the coding gene for at least one is induced somewhat in sporulation, although the genes for these proteins have not been found to be transcribed specifically in the developing spore during sporulation [23–25]. In addition, the genes encoding these proteins are not reported to be transcribed early in spore outgrowth [20]. These proteins have also not been identified in dormant spores [26], although they would be easy to miss since they are generally membrane proteins. Indeed, it seems most probable that the MDR proteins are packaged in the dormant spore, perhaps from synthesis in growing cells or in the sporulating cell prior to the sporulation division, much as are many other proteins essential for the “health” of the germinating spore, one example being the enzymes such as catalases and peroxidases that detoxify a variety of environmental oxidizing agents [1].

A final observation concerns the fact that berberine can inhibit not only *B*. *subtilis* cell growth, but also spore outgrowth, including inhibiting protein synthesis during this period. Since some spore formers could potentially synthesize damaging toxins extremely soon after spore germination, it is possible that berberine might have some advantages in blocking this toxin synthesis and yet still allow spore germination, thus rendering these germinated spores relatively easy to kill [15].

## Supporting Information

S1 Fig DIC image intensities of individual spores germinating at 37°C with L-valine and without berberine (A) or with 200 μg/mL berberine (B). The arrows in panels indicate specific times in germination of a single spore.
(DOCX)

S2 Fig Simultaneous recording of DIC and fluorescence intensities of multiple individual *B*. *subtilis* spores adhered on a coverslip and germinating with 10 mM L-valine and 35 μg/mL berberine in 25 mM K-HEPES buffer. DIC **(A)** and fluorescence **(B)** image intensities as a function of germination time were measured as described in Methods. Normalization of refractility and fluorescence intensities was as described in the legend to Fig 6B. The black arrows indicate the T_1_ times for each individual spore.
(DOCX)

S1 Table Mean values and standard deviations of kinetic parameters of *B*. *subtilis* spore germination with or without 200 μg/mL berberine *. (DOCX)
