# Supplementary material for: Uptake of and Resistance to the Antibiotic Berberine by Individual Dormant, Germinating and Outgrowing Bacillus Spores as Monitored by Laser Tweezers Raman Spectroscopy
Source: PLoS One. 2015 Dec 4;10(12):e0144183. doi: 10.1371/journal.pone.0144183 (PMC4670213; doi:10.1371/journal.pone.0144183)
Supplement: S1 Fig — The arrows in panels indicate specific times in germination of a single spore. (DOCX) [file pone.0144183.s001.docx]

**S1 Fig.**





**S1 Fig. DIC image intensities of individual spores germinating at 37°C with L-valine and without berberine (A) or with** **200** µ**g/mL berberine (B).** The arrows in panels indicate specific times in germination of a single spore.
