# Supplementary material for: Uptake of and Resistance to the Antibiotic Berberine by Individual Dormant, Germinating and Outgrowing Bacillus Spores as Monitored by Laser Tweezers Raman Spectroscopy
Source: PLoS One. 2015 Dec 4;10(12):e0144183. doi: 10.1371/journal.pone.0144183 (PMC4670213; doi:10.1371/journal.pone.0144183)
Supplement: S2 Fig — DIC (A) and fluorescence (B) image intensities as a function of germination time were measured as described in Methods. Normalization of refractility and fluorescence intensities was as described in the legend to Fig 6B. The black arrows indicate the T1 times for each individual spore. (DOCX) [file pone.0144183.s002.docx]

S2 Fig.





**S2 Fig. Simultaneous recording of DIC and fluorescence intensities of multiple individual *B. subtilis* spores adhered on a coverslip and germinating with 10 mM L-valine and 35 µg/mL berberine in 25 mM K-Hepes buffer.** DIC **(A)** and fluorescence **(B)** image intensities as a function of germination time were measured as described in Methods. Normalization of refractility and fluorescence intensities was as described in the legend to Fig 6B. The black arrows indicate the T_1_ times for each individual spore.
