# Supplementary material for: Uptake of and Resistance to the Antibiotic Berberine by Individual Dormant, Germinating and Outgrowing Bacillus Spores as Monitored by Laser Tweezers Raman Spectroscopy
Source: PLoS One. 2015 Dec 4;10(12):e0144183. doi: 10.1371/journal.pone.0144183 (PMC4670213; doi:10.1371/journal.pone.0144183)
Supplement: S1 Table — (DOCX) [file pone.0144183.s003.docx]

**S1Table Mean values and standard deviations of kinetic parameters of *B. subtilis* spore germination with or without 200** µ**g/mL berberine ***

| Treatment | T_1_  (min) | T_lag_  (min) | ∆T_leakage_  (min) | T_release_  (min) | ∆T_release_  (min) | T_lysis_  (min) | Percent  germination in 60 min (No. of germinated spores) |
| --- | --- | --- | --- | --- | --- | --- | --- |
| No berberine | 11.2±6.3 | 15.7±8.4 | 4.4±4.5 | 18.4±8.5 | 2.7±0.9 | 26.4±8.8 | 92%(462) |
| With berberine | 13.6±7.7 | 16.9±7.9 | 3.4±1.8 | 20.0±7.9 | 3.0±0.8 | 27.9±8.3 | 93%(369) |

*PS533 *B. subtilis* spores were germinated at 37°C as described in Methods with 10 mM L-valine in 25 mM K-Hepes buffer (pH 7.4) with or without 200 µg/mL berberine, the germination of > 395 individual spores was followed by DIC microscopy, and kinetic parameters of spore germination were calculated as indicated in Methods.
